# The Hippo transducer TAZ as a biomarker of pathological complete response in HER2-positive breast cancer patients treated with trastuzumab-based neoadjuvant therapy

**DOI:** 10.18632/oncotarget.2449

**Published:** 2014-09-08

**Authors:** Patrizia Vici, Marcella Mottolese, Laura Pizzuti, Maddalena Barba, Francesca Sperati, Irene Terrenato, Anna Di Benedetto, Clara Natoli, Teresa Gamucci, Domenico Angelucci, Maria Teresa Ramieri, Luigi Di Lauro, Domenico Sergi, Monica Bartucci, Rosanna Dattilo, Alfredo Pagliuca, Ruggero De Maria, Marcello Maugeri-Saccà

**Affiliations:** ^1^ Division of Medical Oncology B, Regina Elena National Cancer Institute, Rome, Italy; ^2^ Department of Pathology, Regina Elena National Cancer Institute, Rome, Italy; ^3^ Scientific Direction, Regina Elena National Cancer Institute, Rome, Italy; ^4^ Biostatistics-Scientific Direction, Regina Elena National Cancer Institute, Rome, Italy; ^5^ Department of Experimental and Clinical Sciences, University “G. d’Annunzio”, Chieti, Italy; ^6^ Medical Oncology Unit, ASL Frosinone, Frosinone, Italy; ^7^ Division of Pathology, ‘SS. Annunziata’ Hospital, Chieti, Italy; ^8^ Division of Pathology, ASL Frosinone, Frosinone, Italy; ^9^ Rutgers Cancer Institute of New Jersey, New Brunswick, New Jersey, USA; ^10^ Department of Hematology, Oncology and Molecular Medicine, Istituto Superiore di Sanità, Rome, Italy

**Keywords:** Hippo pathway, TAZ, HER2-positive breast cancer, neoadjuvant therapy, pathological complete response

## Abstract

Activation of the Hippo transducer TAZ is emerging as a novel oncogenic route in breast cancer and it has been associated with breast cancer stem cells. Additionally, TAZ expression has been linked with HER-2 positivity. We investigated the association between TAZ expression and pathological complete response in HER2-positive breast cancer patients treated with trastuzumab-based neoadjuvant therapy. TAZ was assessed in diagnostic core biopsies by immunohistochemistry. To categorize samples with low TAZ and samples with high TAZ we generated a score by combining staining intensity and cellular localization. The pathological complete response rate was 78.6% in patients with low TAZ tumors and 57.6% in patients with high TAZ tumors (p=0.082). In HER2-enriched tumors there was no significant association between TAZ and pathological complete response, whereas in the luminal B subtype the pathological complete response rate was 82.4% in tumors with low TAZ and 44.4% in tumors with high TAZ (p=0.035). This association remained statistically significant when restricting our analysis to triple-positive tumors with expression of both estrogen receptor and progesterone receptor ≥ 50% (p=0.035). Results from this exploratory study suggest that the TAZ score efficiently predicts pathological complete response in Luminal B, HER2-positive breast cancer patients who received neoadjuvant chemotherapy and trastuzumab.

## INTRODUCTION

The Hippo pathway is an evolutionarily conserved regulator of tissue growth [1]. Mutations in Hippo pathway components give rise to tissue overgrowth in flies [2-3], and pathway defects have been associated with tumorigenesis in mice [4]. In human cancer mutations in core genes have rarely been detected in targeted and whole-genome sequencing studies [1]. Nevertheless, altered expression of different effectors has been found in a wide variety of tumors [5], thus suggesting that disruption of the Hippo signaling might result from the crosstalk with other perturbed molecular networks. The main function of Hippo pathway consists in negatively regulating two homologous oncoproteins: the transcriptional co-activator with PDZ-binding motif (TAZ) and Yes-associated protein (YAP). Attenuated Hippo signaling activates TAZ and YAP, which in turn feed a variety of tumor-promoting functions spanning from proliferation and cell survival to epithelial-mesenchymal transition and migration [1]. Moreover, Hippo-independent YAP/TAZ activation has been described [6].

In breast cancer (BC), TAZ has also been linked to cancer stem cells (CSCs) [7, 8], an uncommon subpopulation of cancer cells characterized by increased resistance to therapy-induced death stimuli [9]. Indeed, it has been demonstrated that TAZ sustains self-renewal and tumor-forming ability of breast CSCs [7]. We have recently strengthened this association by using molecularly characterized xenografts generated with patient-derived CSCs and their differentiated counterparts [8]. In an orthotopic mouse model we described the role of TAZ as a mediator of breast CSC metastatic ability and chemoresistance [8]. Moreover, in a preliminary analysis conducted in the clinical setting we found a statistically significant correlation between TAZ expression and shorter disease-free survival in a consecutive series of BC patients, and a positive correlation between TAZ and HER2 positivity [8].

The robustness of our preclinical findings, along with promising early clinical data, prompted this study to explore the association between TAZ, evaluated in diagnostic core biopsies, and pathological complete response (pCR) in HER2-positive BC patients treated with trastuzumab-based neoadjuvant therapy.

## RESULTS

Data on demographics, clinical features, therapy administered and treatment outcomes from 61 HER2-positive BC patients treated with neoadjuvant trastuzumab-based therapy in three Italian Cancer Centers were retrieved from our prospectively maintained database and are illustrated in Table 1.

**Table 1:** Patients’ characteristics

Characteristics	N (%)
**Age at biopsy** Median (range)	48 (31-77)
**Clinical stage** II III	24 (39.3) 37 (60.7)
**Nodal status** Negative Positive	18 (29.5) 43 (70.5)
**Neoadjuvant therapy** EC followed by DT DT followed by ECT	38 (62.3) 23 (37.7)
**Menopausal status** Pre Post	34 (55.7) 27 (44.3)
**Molecular subtype** HER2-enriched Luminal B	26 (42.6) 35 (57.4)

Abbreviations: E: epirubicin; C: cyclophosphamide; D: docetaxel; T: trastuzumab.

To investigate the relationship between TAZ and pCR we generated a TAZ score that takes into account its activation status, as detailed in the methods section. We observed no association between standard clinical-molecular factors and the TAZ score (Table 2), neither did we observe any relationship between standard clinical-molecular factors and pCR (Table 3).

**Table 2:** Association between clinical-molecular factors and TAZ score

Characteristics	TAZ score ≤ 0.50 N (%)	TAZ score > 0.50 N (%)	^■^p-value
**Overall population**	28 (45.9)	33 (54.1)	
**Clinical stage**			
II	9 (37.5)	15 (62.5)	0.289
III	19 (51.4)	18 (48.6)	
**Nodal status**			
Negative	6 (33.3)	12 (66.7)	0.202
Positive	22 (51.2)	21 (48.8)	
**Neoadjuvant therapy**			
EC followed by DT	20 (52.6)	18 (47.4)	0.175
DT followed by ECT	8 (34.8)	15 (65.2)	
**Menopausal status**			
Pre	14 (41.2)	20 (58.8)	0.406
Post	14 (51.9)	13 (48.1)	
**Molecular subtype**			
HER2-enriched	11 (42.3)	15 (57.7)	0.627
Luminal B	17 (48.6)	18 (51.4)	

Abbreviations: E: epirubicin; C: cyclophosphamide; D: docetaxel; T: trastuzumab. ^■^Chi2 test.

**Table 3:** Association between standard clinical-molecular factors and pCR

Characteristics	No pCR N (%)	pCR N (%)	^■^p-value
**Overall population**	20 (32.8)	41 (67.2)	
**Clinical stage**			
II	6 (25.0)	18 (75.0)	0.297
III	14 (37.8)	23 (62.2)	
**Nodal status**			
Negative	7 (38.9)	11 (61.1)	0.511
Positive	13 (30.2)	30 (69.8)	
**Neoadjuvant therapy**			
EC followed by DT	11 (28.9)	27 (71.1)	0.412
DT followed by ECT	9 (39.1)	14 (60.9)	
**Menopausal status**			
Pre	11 (32.4)	23 (67.6)	0.935
Post	9 (33.3)	18 (66.7)	
**Molecular subtype**			
HER2-enriched	7 (26.9)	19 (73.1)	0.4
Luminal B	13 (37.1)	22 (62.9)	

Abbreviations: pCR: pathological complete response; E: epirubicin; C: cyclophosphamide; D: docetaxel; T: trastuzumab. ^■^Chi2 test.

Overall, forty-one (67.2%) patients achieved a pCR (Table 4). In the whole cohort a pCR was recorded in 78.6% of patients with low TAZ tumors and in 57.6% of patients with high TAZ tumors, even though this difference was not statistically significant (p=0.082) (Table 4). Neither the TAZ score nor the selected standard molecular-clinical features showed evidence of a significant impact on pCR in the univariate and multivariate logistic regression models (Supplementary Table 1 and 2).

**Table 4:** Association between the TAZ score and pCR

		No pCR N (%)	pCR N (%)	p-value
**Overall population**		20 (32.8)	41 (67.2)	
	**TAZ score** ≤ 0.50 > 0.50	6 (21.4) 14 (42.4)	22 (78.6) 19 (57.6)	^■^0.082
**HER2-enriched**		7 (26.9)	19 (73.1)	
	**TAZ score** ≤ 0.50 > 0.50	3 (27.3) 4 (26.7)	8 (72.7) 11 (73.3)	^°^0.035
**Luminal B**		13 (37.1)	22 (62.9)	
	**TAZ score** ≤ 0.50 > 0.50	3 (17.6) 10 (55.6)	14 (82.4) 8 (44.4)	^°^0.03
**TP50**		6 (37.5)	10 (62.5)	
	**TAZ score** ≤ 0.50 > 0.50	1 (11.1) 5 (71.4)	8 (88.9) 2 (28.6)	^°^0.03

Abbreviations: pCR: pathological complete response; TP50: triple-positive tumors with expression of estrogen receptor and progesterone receptor ≥ 50%. ^■^Chi2 test, ^°^Fisher Exact test.

**Figure 1:**
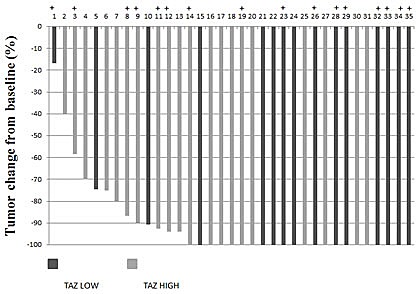
Waterfall plot showing response data for luminal B patients With + are indicated triple-positive tumors with high expression of hormonal receptors (≥ 50%)

When stratifying by molecular subtype, we did not observe any association between TAZ and pCR in HER2-enriched tumors (Table 4). In this subtype, the rate of pCRs was equal between cases with low TAZ and cases with high TAZ. Conversely, in the luminal B subtype the pCR rate was 82.4% in tumors with low TAZ and 44.4% in tumors with high TAZ (p=0.035) (Table 4). The association was confirmed by the regression model (≤ 0.50 vs > 0.50 OR= 5.83; 95% CI, 1.23-27.63; p=0.026; supplementary Table 3). Finally, a statistically significant association was found in the subset of triple-positive tumors with high expression (≥ 50%) of both hormonal receptors (p=0.035) (Table 4). Individual responses in luminal B tumors are illustrated in figure 1.

## DISCUSSION

In this study we evaluated whether an association existed between TAZ and pCR in 61 HER2-positive BC patients who received neoadjuvant chemotherapy plus trastuzumab. To our knowledge, this is the first study investigating the predictive role of the Hippo transducer TAZ in this setting. No statistically significant association between TAZ and pCR was reported in the entire cohort. However, an association emerged when stratifying by molecular subtype. While TAZ did not have any impact on pCR in HER2-enriched tumors, a significant association was observed in Luminal B tumors and in the subset with high expression of both hormonal receptors. As we did not note any association between standard clinical-molecular features and pCR, we provided hints supporting the hypothesis that TAZ is an independent predictive factor for pCR. Indeed, the inclusion of the proliferation marker Ki-67 in the models did not affect our study results (data available upon request).

In interpreting our results, we are aware that this study is limited by its retrospective nature and the relatively restricted number of patients evaluated. Nevertheless, our study has some important strengths. First, the experimental path underlying this study deserves to be mentioned. Patient-derived xenografts generated with CSCs allow to reconstitute human tumors in the murine background and to amplify them through serial transplantation [10, 11]. As mentioned above, our interest on TAZ stemmed from molecularly characterized patient-derived xenografts obtained with the delivery of mammospheres, which are enriched for CSCs, generated from patients with Luminal B tumors [8]. Thus, our preclinical model may represent a promising tool for streamlining the preclinical-clinical transition of novel biomarkers.

Second, over the past decade many prognostic and predictive multigene classifiers have been developed to assist in clinical decision-making [12]. Notwithstanding, even the broadest investigated genomic assays originally developed to foresee long-term outcomes are struggling to gain widespread consensus due to the unclear gain in precision and cost-effectiveness over standard prognostic factors [13]. We developed a clinically applicable assay with the potential to expand the current pipeline of biomarkers used in routine clinical practice. From a biological standpoint, it is worth mentioning that the TAZ score employed in this study was generated by considering cellular localization, and consequently TAZ activation. Indeed, cytoplasmic accumulation reflects ubiquitin-mediated proteolysis, whereas nuclear translocation is usually interpreted as an activation of the TAZ-mediated oncogenic transcriptional program [14].

As pCR in an established surrogate marker for better long-term outcomes [15], it is of utmost importance to identify biomarkers of response and resistance to HER2-directed therapy. Even though a variety of putative predictive biomarkers have been proposed, spanning from gene expression profiles to mutations in HER2 and genes belonging to its transduction machinery [16-19], we are still waiting for validation studies. Even considering the sample size of our study, the magnitude of the difference in the pCR rate seen in Luminal B tumors according to TAZ status is rather uncommonly reported, and in our opinion represents a background for a prospective study with biomarker validation purposes. Moreover, the lesson we learned from clinical trials, such as NeoALTTO [20], GEPARQUINTO [21], and NeoSphere [22], is that hormone receptor-positive tumors achieve a lower rate of pCR compared with hormone receptor-negative tumors. Thus, foreseeing pCR after anti-HER2-based neoadjuvant therapy acquires an even more relevant therapeutic implication in this setting. With the present analysis we have provided clues on the potential predictive value of a TAZ-based biomarker in HER2-positive and hormone receptor-positive tumors.

In summary, the message conveyed by this study is that the TAZ score we developed appears extremely promising in predicting the individual likelihood to achieve a pCR to trastuzumab-based neoadjuvant therapy in Luminal B, HER2-positive BC patients. As a consequence, we plan to prospectively validate the TAZ score as a molecular predictor in this BC subtype. Finally, our goal is to examine core components of the Hippo pathway in different therapeutic settings and molecular subtypes with the aim of identifying Hippo pathway-related prognostic and predictive biomarkers.

## METHODS

### Study Participants and procedures

Included patients were 61 HER2-positive BC patients who received neoadjuvant trastuzumab-based therapy. Patients were considered suitable for inclusion if they had received trastuzumab as a part of their neoadjuvant treatment, had complete data on baseline clinical features, therapy administered and treatment outcomes, if the planned treatment was completed, and the amount of biological materials was sufficient for TAZ analysis in their biopsies. Patients were treated with two different schedules: epirubicin 120 mg/m^2^ plus cyclophosphamide 600 mg/m^2^ administered intravenously (IV) on day 1 every 21 days for four cycles followed by docetaxel 100 mg/m^2^ plus trastuzumab 6 mg/kg (after a loading dose of 8 mg/kg) administered IV on day 1 every 3 weeks for four cycles, or a reverse sequence with the administration of trastuzumab for the whole length of chemotherapy. pCR was defined as no residual invasive tumor in both breast and axilla (ypT0/is ypN0), and was assessed by local pathologists. The immunohistochemical assessment of estrogen receptor (ER), progesterone receptor (PgR) and HER2 was performed in formalin-fixed paraffin-embedded tissues using the monoclonal antibodies (MoAbs) 6F11, 1A6 (Menarini, Florence, Italy) and the polyclonal antibody A0485 (PoAb, Dako, Milan, Italy). TAZ was evaluated with the MoAb anti-TAZ (M2-616, BD Pharmingen, San Jose, CA,USA). ER and PgR were considered positive when ≥1% of the neoplastic cells showed a distinct nuclear immunoreactivity. HER2 positivity was defined, according to ASCO-CAP guidelines, as 3+ overexpression by immunohistochemical testing or as 2+ with HER2 amplification by silver in situ hybridization (SISH, Inform HER2 DNA Probe; Inform Chr17 probe, Roche Diagnostics, Milan, Italy) [23]. TAZ was evaluated in diagnostic core biopsies and considered positive when >10% of tumor cells displayed nuclear and/or cytoplasmic immunostaining. TAZ staining intensity was graded on a four-grade scale (0: negative, 1+: weak, 2+: moderate, 3+: strong). The TAZ score was obtained by multiplying the staining intensity × 1.5 (nuclear localization) or 0.5 (cytoplasmic localization), in order to take into account its activation status. Using the median score of all tumors as a cut-off point, tumors with a score ≤0.5 were considered as TAZ low, whereas tumors with a score >0.5 as TAZ high. TAZ expression and localization were independently evaluated by two investigators (MM and ADB) who were masked to treatment outcome. Discordant cases were reviewed at a face-to-face meeting.

### Statistical analysis

We computed descriptive statistics for all variables of interest. Continuous data were reported as mean and standard deviation and we represented categorical data with frequencies and percentage values. In order to assess the relationships between categorical variables, different tests were employed: the Pearson’s Chi-squared test of independence (2-tailed) and the Fisher Exact test. We used a univariate logistic regression model to identify variables that could influence the study outcome (pCR). We planned to insert variables significant in the univariate analysis in a multivariate logistic regression. We considered statistically significant p values less than 0.05. Statistical analyses were carried out using SPSS software (SPSS version 21, SPSS Inc., Chicago, IL, USA).

## SUPPLEMENTARY TABLES


