# Supplementary material for: The Hippo transducer TAZ as a biomarker of pathological complete response in HER2-positive breast cancer patients treated with trastuzumab-based neoadjuvant therapy
Source: Oncotarget. 2014 Sep 8;5(20):9619–25. doi: 10.18632/oncotarget.2449 (PMC4259424; doi:10.18632/oncotarget.2449)
Supplement: Supplementary file 1 [file oncotarget-05-9619-s001.pdf]

The Hippo transducer TAZ as a biomarker of pathological complete response in HER2-positive breast cancer patients treated with trastuzumab-based neoadjuvant therapy

Supplementary Material

Supplementary Table 1. Univariate regression model for pCR

Characteristics	OR	95%CI	P-VALUE
**Clinical stage**			
II	1		
III	0.55	0.17-1.71	0.300
**Nodal status**			
Negative	1		
Positive	1.47	0.46-4.64	0.512
**Neoadjuvant therapy**			
EC followed by DT	1		
DT followed by ECT	0.63	0.21-1.89	0.413
**Menopausal status**			
Pre	1		
Post	0.96	0.33-2.80	0.935
**Molecular subtype**			
HER2-enriched	1		
Luminal B	0.62	0.21-1.88	0.400
**TAZ score**			
> 0.50	1		
≤ 0.50	2.70	0.87-8.42	0.086

Supplementary Table 2. Multivariate regression model for pCR

Characteristics	OR	95%CI	P-VALUE
**Clinical stage**			
II	1		
III	0.22	0.04-1.21	0.082
**Nodal status**			
Negative	1		
Positive	3.66	0.63-21.21	0.148
**Neoadjuvant therapy**			
EC followed by DT	1		
DT followed by ECT	0.64	0.17-2.36	0.503
**Menopausal status**			
Pre	1		
Post	0.53	0.14-1.98	0.344
**Molecular subtype**			
HER2-enriched	1		
Luminal B	0.45	0.13-1.60	0.221
**TAZ score**			
> 0.50	1		
≤ 0.50	3.12	0.90-10.84	0.073

Supplementary Table 3. Univariate regression model for pCR stratified by molecular subtype.

Characteristics		OR	95%CI	P-VALUE
**Molecular subtype**				
Luminal B				
	**TAZ score**			
	> 0.50	1		
	≤ 0.50	5.83	1.23-27.63	0.026
HER2 subtype				
	**TAZ score**			
	> 0.50	1		
	≤ 0.50	0.97	0.17-5.59	0.973
